# Distinct mucosal microbial communities in infants with surgical necrotizing enterocolitis correlate with age and antibiotic exposure

**DOI:** 10.1371/journal.pone.0206366

**Published:** 2018-10-26

**Authors:** Joann Romano-Keeler, Meghan H. Shilts, Andrey Tovchigrechko, Chunlin Wang, Robert M. Brucker, Daniel J. Moore, Christopher Fonnesbeck, Shufang Meng, Hernan Correa, Harold N. Lovvorn, Yi-Wei Tang, Lora Hooper, Seth R. Bordenstein, Suman R. Das, Jörn-Hendrik Weitkamp

**Affiliations:** 1 Department of Pediatrics, Vanderbilt University, Nashville, Tennessee, United States of America; 2 Department of Medicine, Vanderbilt University Medical Center, Nashville, Tennessee, United States of America; 3 Research Bioinformatics, Medimmune, Gaithersburg, Maryland, Tennessee, United States of America; 4 Genome Technology Center, Stanford University, Palo Alto, California, United States of America; 5 Department of Biological Sciences, Vanderbilt University, Nashville, Tennessee, United States of America; 6 Department of Pathology, Microbiology & Immunology, Vanderbilt University, Nashville, Tennessee, United States of America; 7 Vanderbilt Institute for Infection, Immunology and Inflammation, Vanderbilt University Medical University, Nashville, Tennessee, United States of America; 8 Department of Biostatistics, Vanderbilt University, Nashville, Tennessee, United States of America; 9 Department of Pediatric Surgery, Vanderbilt University, Nashville, Tennessee, United States of America; 10 Department of Laboratory Medicine, Memorial Sloan Kettering Cancer Center, New York, New York, United States of America; 11 Department of Immunology, The University of Texas Southwestern Medical Center, Dallas, Texas, United States of America; 12 Department of Biological Sciences, Vanderbilt University, Nashville, Tennessee, United States of America; Arizona State University, UNITED STATES

## Abstract

**Objective:**

Necrotizing enterocolitis (NEC) is the most common surgical emergency in preterm infants, and pathogenesis associates with changes in the fecal microbiome. As fecal samples incompletely represent microbial communities in intestinal mucosa, we sought to determine the NEC tissue-specific microbiome and assess its contribution to pathogenesis.

**Design:**

We amplified and sequenced the V1-V3 hypervariable region of the bacterial 16S rRNA gene extracted from intestinal tissue and corresponding fecal samples from 12 surgical patients with NEC and 14 surgical patients without NEC. Low quality and non-bacterial sequences were removed, and taxonomic assignment was made with the Ribosomal Database Project. Operational taxonomic units were clustered at 97%. We tested for differences between NEC and non-NEC samples in microbiome alpha- and beta-diversity and differential abundance of specific taxa between NEC and non-NEC samples. Additional analyses were performed to assess the contribution of other demographic and environmental confounding factors on the infant tissue and fecal microbiome.

**Results:**

The fecal and tissue microbial communities were different. NEC was associated with a distinct microbiome, which was characterized by low diversity, higher abundances of *Staphylococcus* and *Clostridium_sensu_stricto*, and lower abundances of *Actinomyces* and *Corynebacterium*. Infant age and vancomycin exposure correlated with shifts in the tissue microbiome.

**Conclusion:**

The observed low diversity in NEC tissues suggests that NEC is associated with a bacterial bloom and a distinct mucosal bacterial community. The exact bacterial species that constitute the bloom varied by infant and were strongly influenced by age and exposure to vancomycin.

## Introduction

Necrotizing enterocolitis (NEC) is a common and frequently fatal intestinal complication in premature infants [1,2]. Experiments in germ-free animals and toll-like receptor targeted knock out mice strongly suggest a bacterial antigen is critical for the initiation of intestinal inflammation and NEC development [3–6]. Bacterial DNA is present in larger quantities in acute human NEC specimens compared to samples collected after NEC has clinically resolved [7]. A number of different gram-positive and gram-negative bacteria as well as viruses have been associated with NEC [8]. Indeed, microbial community studies using 16S rRNA gene sequencing of the fecal microbiome demonstrate a reduction in microbial community diversity with a shift towards potentially pathogenic subgroups [9–12].

We previously detected significant differences in the microbiome between surgical tissue and parallel collected fecal samples in preterm infants without NEC [13]. We hypothesized the existence of a specific microbial profile at the site of injury in the small intestinal mucosa of premature infants with NEC that has not been previously recognized in fecal microbiome studies. Hence, we sought to interrogate differences in the tissue-level and fecal microbiomes in infants with and without NEC to determine bacterial communities at the site of injury and their representation in feces. As intestinal tissue cannot ethically be collected from healthy infants, we included infants with intestinal diseases other than NEC in this study for comparison. We detected a statistically significant increase in the abundance of *Staphylococcus* and *Clostridium_sensu_stricto* in NEC compared to non-NEC tissue samples when controlling for age and history of antibiotic exposure.

## Materials and methods

### Ethics statement

This study was approved by the Vanderbilt University Institutional Review Board (protocol number 090161). All infants hospitalized at the Monroe Carell Jr. Children’s Hospital at Vanderbilt were eligible for the study if they underwent intestinal resection at <180 days of age. We obtained written informed consent from parents, the next of kin, caretakers, or guardians on behalf of the minors/children enrolled in the study to permit collection of tissue and metadata from the medical records including gestational age, birth weight, race, sex, mode of delivery, maternal or fetal indications for delivery, antibiotic exposure, enteral feeding regimens, diagnoses and type of surgical resection.

### Sample collection

Tissue collected at the time of surgery was gently rinsed with sterile saline solution, and immediately cryopreserved in sterile containers [13]. Fecal material was collected by either taking the patient’s first post-operative stool or by scraping surgical tissue; samples were immediately cryopreserved (**Table 1**). The clinical and intraoperative diagnosis of NEC was confirmed by a pediatric pathologist after histologic examination of the resected specimen and by review of the operative and surgical pathology reports.

**Table 1:** Clinical characteristics of patient samples.

Sample ID	Indication for intestinal resection	Gestational age (wks)	Birth weight (g)	Sex	Age at surgery (d)	Tissue type	Mode of delivery	Feeding	Preoperative antibiotics (d)*	Antibiotic type†	Sample types included in analysis
N4	NEC	26	780	F	6	Ileum	Vaginal	NPO	2	A, G, V, MZ	Fecal, tissue
N6	NEC	29	1,630	M	46	Colon	Vaginal	NPO	22	V, G, P	Fecal, tissue
N9	NEC	28	850	M	38	Jejunum	C-section	EBM	2	A, G	Fecal, tissue
N20	NEC	33	1,740	M	5	Colon	Vaginal	Formula	5	A, G	Tissue
N27	NEC	25	440	F	27	Ileum	C-section	EBM	1	V, G, M	Fecal^‡^, tissue
N33	NEC	30	1,583	F	7	Ileum	C-section	Formula	6	A, G, V, C, MZ	Fecal, tissue
N34	NEC	30	1,550	M	9	Jejunum	Vaginal	Formula	3	A, G, V, MZ	Fecal, tissue
N37	NEC	25	650	F	8	Ileum	C-section	EBM	3	A, G, V	Fecal, tissue
N39	NEC	33	2,101	F	8	Ileum	Vaginal	Formula	6	A, G, MZ	Fecal, tissue
N324	NEC	30	1,420	F	11	Ileum	C-section	Formula	1	A, G, V, P	Tissue
C22	Spontaneous perforation	25	850	M	6	Ileum	Vaginal	NPO	6	A, G, MZ	Fecal, tissue
C23	Spontaneous perforation	24	650	F	1	Ileum	Vaginal	NPO	1	A, G, MZ, M	Fecal
C18	Congenital volvulus	31	1,400	F	1	Ileum	C-section	NPO	1	A, G	Fecal, tissue
C28	Congenital volvulus	32	2,590	F	0	Ileum	C-section	NPO	0	A, G, MZ	Fecal, tissue
C17	Mesenteric ischemia	26	700	M	6	Jejunum	C-section	EBM	6	A, G	Fecal, tissue
C5	Intestinal atresia	35	2,605	M	3	Ileum	Vaginal	NPO	2	A, G	Fecal^‡^, tissue
C8	Intestinal atresia	34	2,015	F	5	Jejunum	Vaginal	NPO	5	A, G	Fecal, tissue
C24	Stricture removal	28	1,268	M	57	Jejunum	Vaginal	Formula	17	A, G, CL, V	Fecal, tissue
C26	Stricture removal	29	1,664	M	43	Ileum	C-section	NPO	18	A, G, CL, V	Fecal, tissue
C19	Stricture removal	39	3,454	F	132	Colon	Vaginal	NPO	131	A, G, C, CP, CT, M, V	Fecal, tissue
C16	Hirschsprung’s disease	36	2,793	M	4	Colon	Vaginal	NPO	0	None	Tissue
C14	Re-anastomosis	27	830	F	60	Ileum	Vaginal	Formula	14	A, G, C, MZ, V	Fecal, tissue
C15	Re-anastomosis	26	790	F	65	Ileum	C-section	Formula	12	A, G, MZ, P, V	Fecal, tissue
C21	Re-anastomosis	32	1,660	M	57	Ileum	Vaginal	Formula	24	A, G, M, V, T, CL	Fecal, tissue

* 0, less than 24 hours

^†^ A, ampicillin; G, gentamicin; V, vancomycin; C, cefotaxime; CL, clindamycin; CP, cefepime; CT, ceftriaxone; M, meropenem; P piperacillin-tazobactam; MZ, metronidazole; T, tobramycin

^‡^ Feces adherent to collected mucosa; all other fecal samples collected at patient’s first post-operative stool

NEC, necrotizing enterocolitis; NPO, nil per mouth; EBM, expressed breast milk; DBM, donor breast milk

### DNA extraction and amplification of 16S rRNA gene

We extracted DNA from fresh NEC and non-NEC surgical tissue and corresponding fecal samples as previously described [13]. Briefly, we extracted DNA from 15–25 mg of intestinal tissue and 180–200 mg of feces and amplified the V1-V3 hypervariable region of bacterial 16S rRNA with previously validated primers: 5F (5’-TGGAGAGTTTGATCCTGGCTCAG-3’) and 532R (5’-TACCGCGGCTGCTGGCAC-3’) [14]. PCR was conducted as described [13] and barcoded amplicons were gel purified (Qiagen), quantified, and pooled prior to sequencing on a 454 FLX Titanium sequencer. Sequencing negative controls—template-free sterile water, processed with the same DNA extraction and PCR amplification kits as the real samples—were sequenced on the same run [15].

### Pyrosequencing and data analysis

Sequences generated from the pyrosequencing of barcoded 16S rRNA gene PCR amplicons were analysed using mothur (http://mothur.org) [16] by following the 454 SOP as of 13 March 2017. Sequences were aligned to the SILVA database release 123 [17] and taxonomically classified with the Ribosomal Database Project (RDP) classifier 11 [18]. Chimeric sequences as detected by UCHIME were removed [19]. OTUs were clustered at 97% similarity. Prior to statistical analysis, samples with <400 reads were discarded (N = 3).

Phylogenetic Investigation of Communities by Reconstruction of Unobserved States (PICRUSt) was used to predict metagenomic and functional composition of the samples from 16S rRNA sequences [20]. Prior to PICRUSt analyses, closed reference OTUs were picked against the GreenGenes database 13_5 [21] using uclust [22] in QIIME 1.9.1 [23]; taxonomy assignments were made using the RDP Classifier 2.2 [24]. Functions of genes were assigned using the KEGG Orthology database [25].

Statistical analysis was performed in R using MGSAT (https://bitbucket.org/andreyto/mgsat), which wraps a number of R packages, including *vegan* [26] to perform alpha- and beta- diversity analyses and *DESeq2* [27], *GeneSelector* [28], and *stabsel* [29] for testing taxonomic associations with metadata. When testing taxonomic associations with metadata, we report the q-values computed with the Benjamini & Hochberg false discovery rate method to adjust for multiple comparisons [30].

For diversity and richness estimates, full count matrices as produced by the mothur annotation were used [31]. To compare microbial alpha diversity estimates between groupings, we estimated Shannon-Wiener (H') and Simpson’s diversity indices; to compare microbial richness estimates, we estimated observed OTUs and calculated S. chao1 estimates. Counts were rarefied to the lowest library size of all the samples (number of reads per sample = 445), and then abundance-based and incidence-based alpha diversity indices and richness estimates were computed. This was repeated multiple times (*n* = 400), and the results were averaged. Incidence-based estimates were computed on pools of observations split by the relevant metadata attribute, and in each repetition, observations were also stratified to balance the number of observations at each level of the metadata attribute. Inverted Simpson and Shannon diversity indices were converted into corresponding Hill numbers [32]. Linear models were fit to test for associations between abundance-based richness and diversity estimates and metadata attributes.

We applied the PermANOVA (permutation-based analysis of variance) [33] test of statistical significance (as implemented in the *Adonis* function of the *R vegan* package) [34] on the association between the abundance profile dissimilarities and the metadata variables. We used the Bray-Curtis dissimilarity index [35] and 4000 permutations. The counts were normalized to simple proportions within each observation.

When differential abundance analysis was performed, in order to remove the likely non-informative features and to reduce the associated penalty from the multiple testing correction applied after univariate tests, we used unbiased metadata-independent filtering at each taxonomy level by eliminating all taxa that were detected with a mean proportional abundance of less than 0.0005. The absolute counts from the removed features were aggregated into a category “other,” which was taken into an account when computing simple proportions during data normalization, but were otherwise discarded. When testing taxonomic associations with metadata, for each feature, we also obtained, from the same test done on the full dataset, the p-value computed using the test implementation from R *exactRankTests* package [36], the q-value computed with the Benjamini & Hochberg false discovery rate method in the package function *p*.*adjust* [37], and several types of the effect size such as common language effect size and rank biserial correlation [38]. To evaluate the influence of confounders, models were built in DESeq2 with pre-selected covariates added in.

*Stabsel* is a stability selection approach implemented in the R package *stabs* [29]. This feature selection method implements a stability selection procedure described in [39] with the improved error bounds described in [40]. Elastic net (from R package *glmnet* [41]) was used as the base feature selection method that was wrapped by the stability protocol. For groupings with two factor levels, a binomial family model was built with the grouping as a response and the matrix of the abundance values as predictors. The mixing parameter α of *glmnet* was selected based on a 15-fold cross-validation minimizing deviance on the full dataset. The predictors were first normalized to simple proportions within each multivariate observation, transformed with the inverse hyperbolic sign $\log\left(x+\surd (x^{2}+1)\right)$, and then standardized to zero means and unit variances. With its multivariate base feature selection method, this protocol can potentially detect those correlated groups of biologically relevant features that will be missed by the univariate methods. The ranking of taxa and their probability of being selected into the model were reported, as well as the probability cutoff corresponding to the per-family error rate (PFER) that is controlled by this method. Our PFER cutoff was set to 0.05, and the target number of features selected by the base classifier was set to $\sqrt{(0.8}\times p)$ where *p* is the total number of features (39). In our experience with omics datasets, the PFER control in this method is fairly conservative, and we typically look at the ranking of features as opposed to only concentrating on features that pass the PFER cutoff.

### Data deposition

All sequences reported in this paper have been deposited into the NCBI sequence short read archive (accession no. SRR7993700-SRR7993745).

## Results

### Demographic and antimicrobial exposure characteristics were similar between NEC and non-NEC infants

We collected and analyzed fresh surgical tissue and corresponding fecal samples from 10 patients with NEC and 14 patients without NEC; in total, 44 samples were analyzed (fecal N = 21; tissue N = 23) (**Table 1**). Surgical samples included patients with spontaneous intestinal perforations, ileal and jejunal atresias, midgut volvulus, and mesenteric ischemic bowel injuries. Mean gestational age, birth weight and postnatal age were 29 weeks (range 25–33 weeks), 1,274 grams (range 440–2,101 grams), and 17 days (range 5–46 days) for NEC infants and 30 weeks (range 24–39 weeks), 1,662 grams (range 650–3,454 grams), and 31 days (0–132 days) for non-NEC patients, respectively (all t-tests p>0.05). Female infants represented 60% and 50% of the study population in the NEC and non-NEC groups, respectively. Except for two colon samples among the non-NEC group and two colon samples within the NEC group, all analyzed tissues were from the ileum or jejunum. For the non-NEC group, one fecal sample (C5) was adherent to the mucosa when collected, for the NEC group there was one (N27). All but one infant from the non-NEC group had perinatal antibiotic exposure. Mean number of antibiotic exposure days prior to surgery were less in the NEC group (5 days, range 1–22) compared to the non-NEC group (17 days, range 0–131) but means were not statistically different (t-test with Welch’s correction, p = 0.180). Both the NEC and non-NEC groups contained infants receiving breast milk, infant formula, or no enteral nutrition prior to sample collection. Of non-NEC infants, 36% were delivered via C-section compared to 50% of infants in the NEC group.

### Microbial diversity was reduced in NEC samples compared to non-NEC samples

After quality filtering and removal of chimeras and non-bacterial sequences, barcoded 16S rRNA amplicons generated a total of 59,778 sequences for fecal and 72,791 sequences for tissue samples. The mean (range) number of fecal sample sequences was 4,719 (697–15,319) for NEC and 2,510 (589–6,530) for non-NEC subjects, and the mean (range) number of tissue sample sequences was 2,322 (445–6,066) sequences for NEC and 2,799 (634–7,906) for non-NEC subjects.

**Fig 1:**
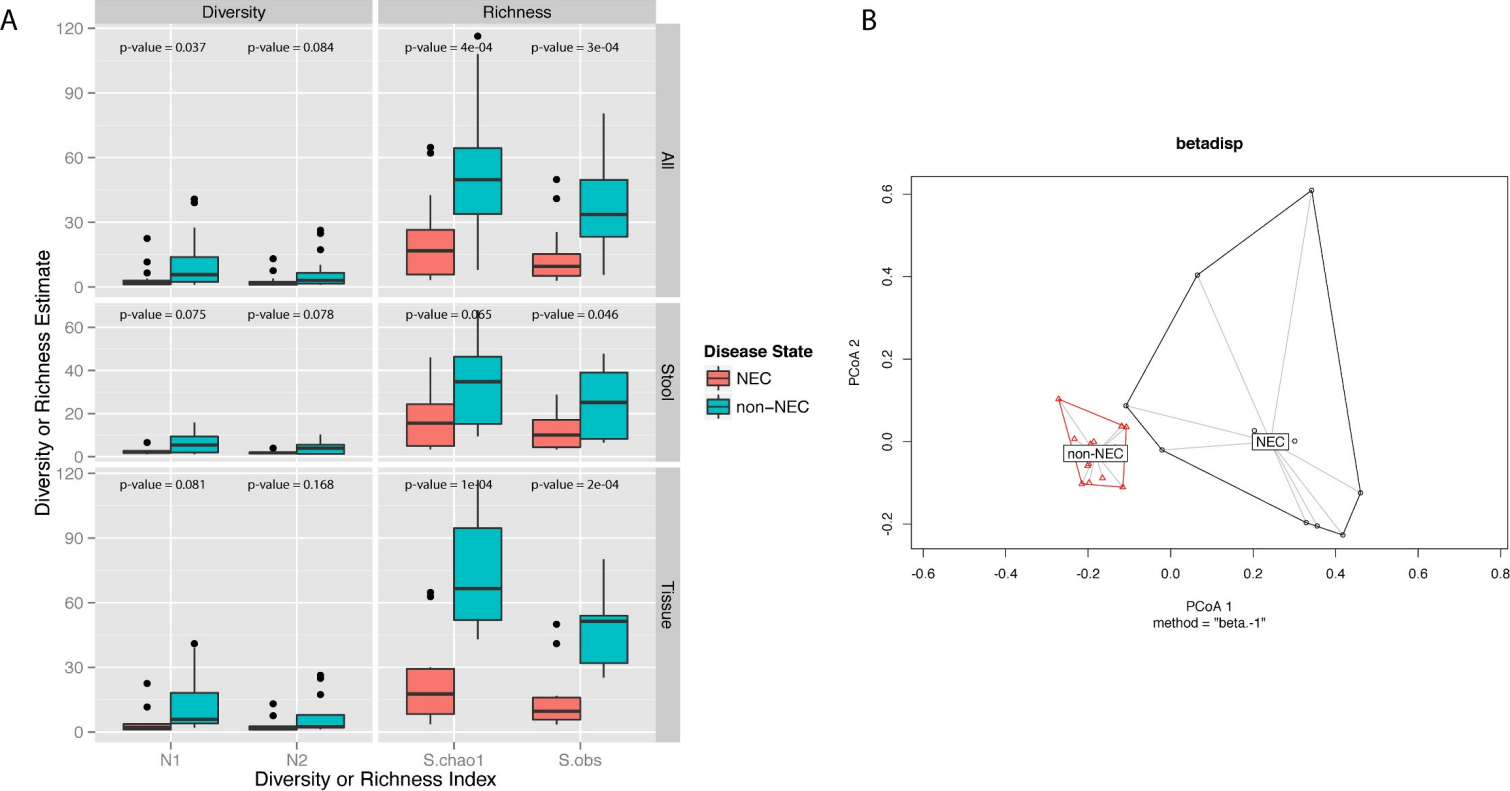
**A)** Boxplots of tissue microbial diversity and richness in infants with and without necrotizing enterocolitis (NEC) at the operational taxonomic unit (OTU) level for all samples, stool alone, and tissue alone. After rarefaction to the lowest library size of all the samples (445 reads per sample), α diversity and richness estimates were calculated per each sample. This process was repeated 400 times and results were averaged. The Shannon and inverse Simpson indices were calculated to estimate abundance-based OTU diversity, while the Chao1 estimator and observed taxa counts were calculated to estimate abundance-based OTU richness. Displayed p-values were obtained after testing for a non-zero coefficient of a normal linear model that used NEC/non-NEC group membership as predictor of richness or diversity. All tested richness and diversity indices for both tissue and stool samples were at or near significantly lower in NEC compared to non-NEC samples. **B)** Principal coordinates analysis (PCoA) plot of tissue samples, labelled by NEC status. Bray-Curtis dissimilarities between samples were calculated at the genus level after normalizing read counts to simple proportions and after rarefaction to the lowest library size (445 reads per sample). The centroids between the NEC and non-NEC samples were significantly dissimilar (*Adonis* PerMANOVA p-value = 0.0002).

Prior to estimating microbial alpha-diversity or richness, samples were rarefied to the lowest library size of all the samples (445 reads per sample). When testing for a non-zero coefficient of a normal linear model that used NEC/non-NEC group membership as predictor of richness, microbial richness and diversity were lower in NEC samples compared to non-NEC samples (**Fig 1A**). In tissue samples, when comparing microbial richness or diversity in tissue from NEC and non-NEC subjects, p-values for all tested richness estimates were <0.05 and there was a trend towards lower alpha diversity estimates in tissue from infants with NEC compared to those without NEC (p-values: N1 = 0.081, N2 = 0.168) (**Fig 1A**). Both microbial richness (observed OTU counts (S.obs) p-value = 0.046, S.Chao1 p-value = 0.065) and alpha diversity (N1 p-value = 0.075, N2 p-value = 0.078) were at or near significantly lower in stool from infants with NEC compared to those without (**Fig 1A**).

### NEC and non-NEC samples exhibited distinct microbial profiles

**Fig 2:**
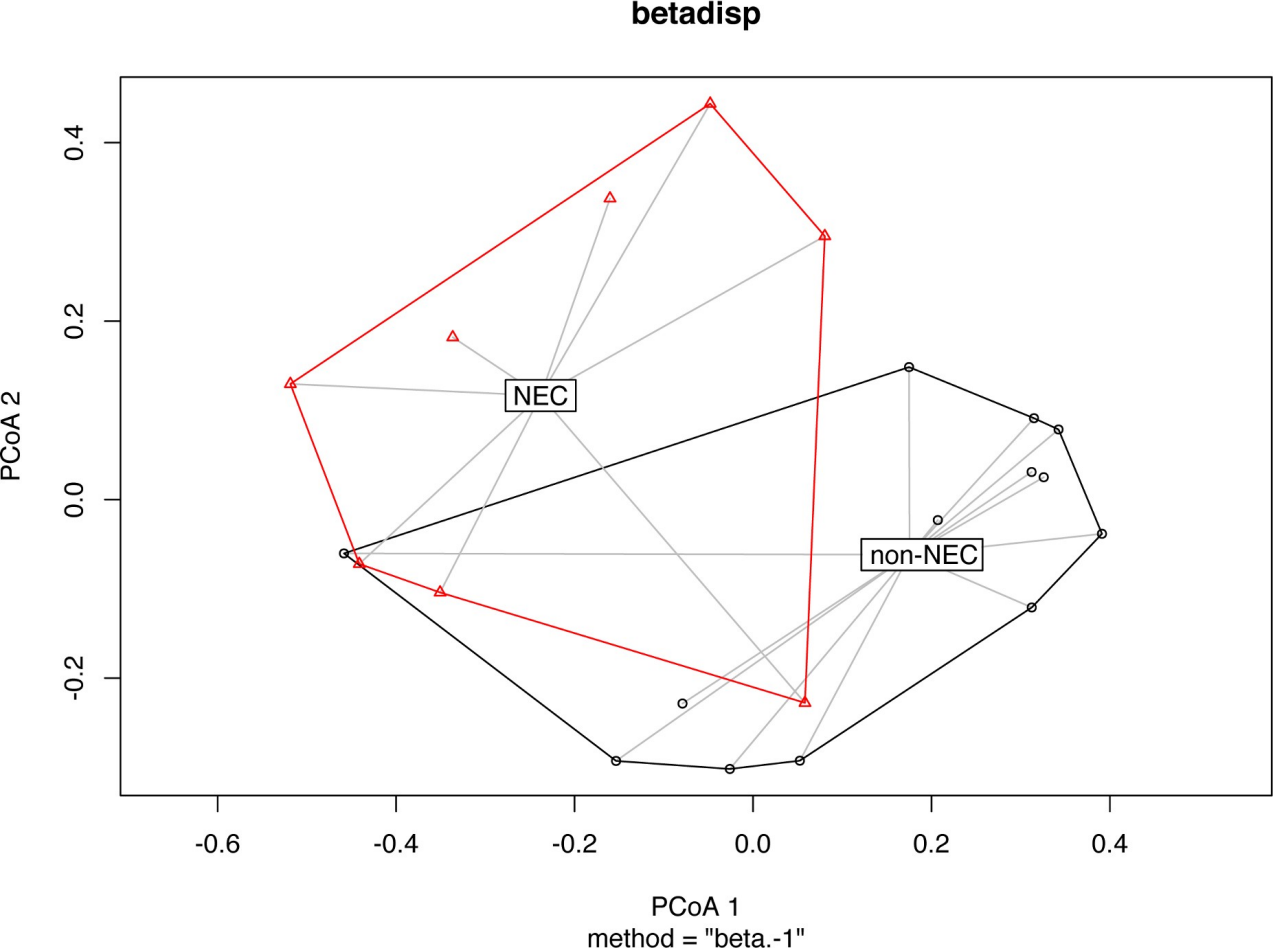
Principal coordinates analysis (PCoA) plots of stool samples, labelled by necrotizing enterocolitis (NEC) status. Bray-Curtis dissimilarities between samples were calculated at the genus level after normalizing read counts to simple proportions. The centroids between the NEC and non-NEC samples were significantly dissimilar (*Adonis* PerMANOVA p-value = 0.003).

Prior to estimating beta diversity, samples were rarefied to the lowest library size (445 reads/sample). Principal Component Analysis (PCoA) using pairwise Bray-Curtis dissimilarities demonstrates distinct microbial genus composition of tissue samples from NEC versus non-NEC patients (*Adonis* test p-value = 0.0003) (**Fig 1B)**. The microbial communities isolated from NEC and non-NEC fecal samples were also significantly dissimilar (**Fig 2,** Bray-Curtis dissimilarities calculated at the genus level, *Adonis* test p-value = 0.003). In contrast to the more uniform pattern in non-NEC tissues, microbial composition in NEC tissue clustered in separate coordinates indicating discrete colonization types.

**Fig 3:**
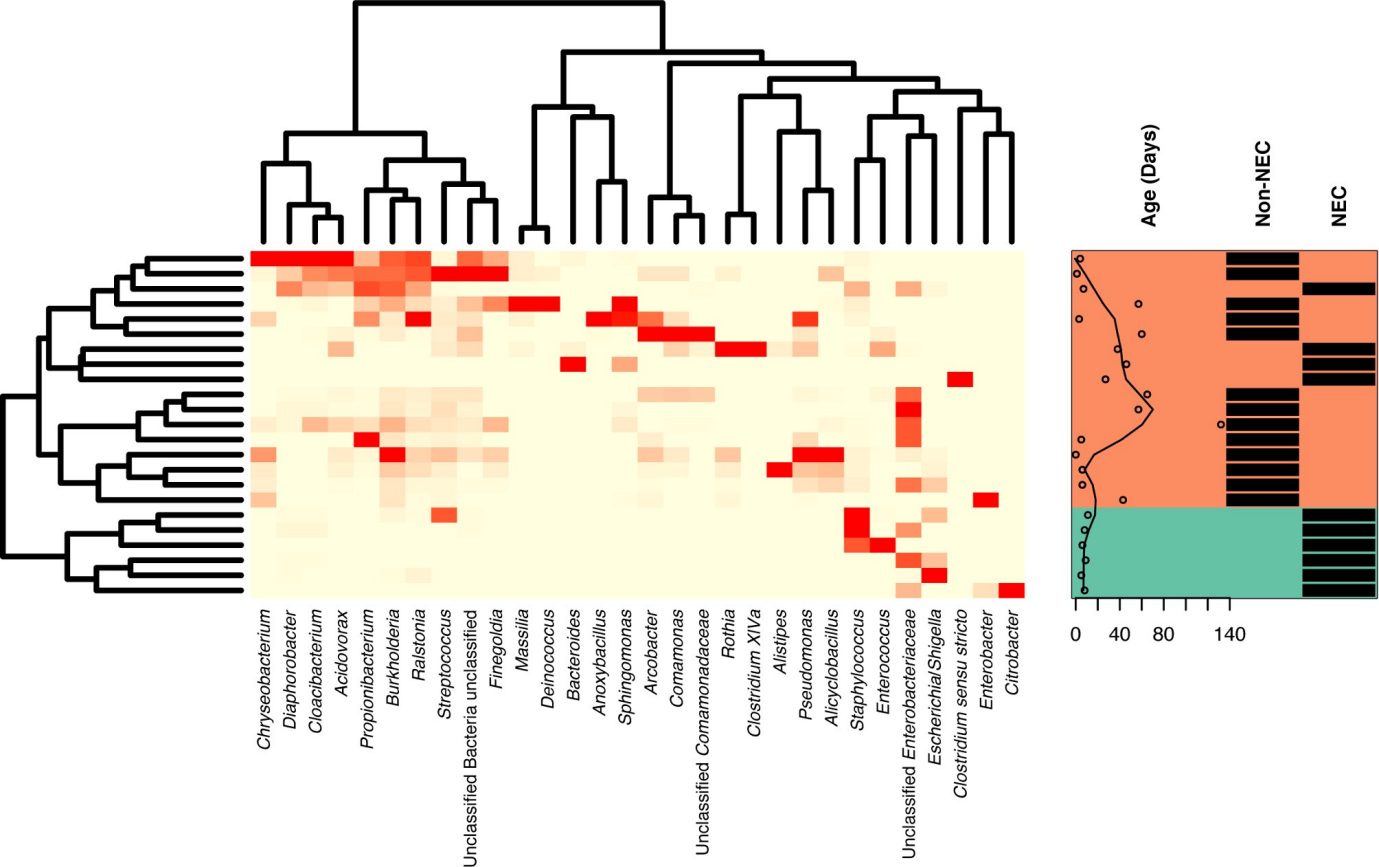
Heatmap of microbial abundance profiles of infant gut tissue at the genus level. The 30 most abundant genera are shown. Infant age, in days, and necrotizing enterocolitis (NEC) status are labelled for each sample. Clustering due to NEC status can be observed.

**Fig 4:**
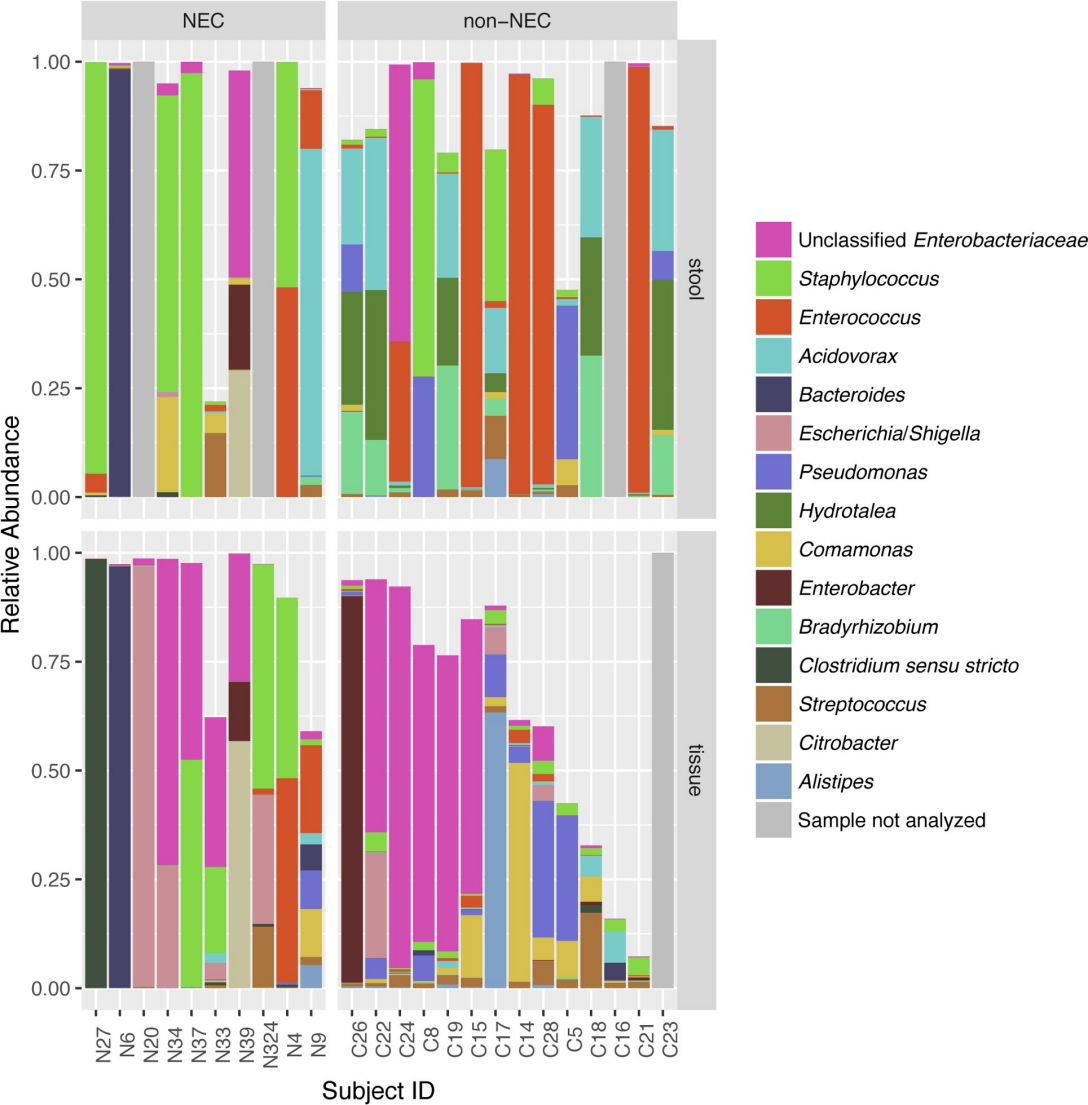
Stacked bar graph showing genus level taxonomic composition for each individual sample, expressed as a proportion of reads. Infant sample ID is on the x-axis. The top 15 genera with the highest average relative abundance are shown. Samples are stratified by necrotizing enterocolitis (NEC) status, and whether sample was tissue or stool. Tissue and stool samples from the same infant had dissimilar microbial profiles.

Specific taxa associated with the differential microbial profiles of NEC and non-NEC samples. **Fig 3** shows a heatmap of the top 30 most abundant genera found in tissue samples across the bottom, with sample clustering on the left and each individual sample marked on the right with both infant age in days at time of collection and whether the sample was from an infant with or without NEC. NEC and non-NEC samples generally formed two distinct clusters. Bacterial genus level assignments for tissue and fecal samples comparing NEC with non-NEC patients are depicted in **Fig 4**; NEC tissue samples were more likely to be dominated by a single genus (**Fig 1A**), including *Staphylococcus*, *Clostridium*, *Escherichia*, or *Bacteroides* than non-NEC samples. Stool and tissue communities were significantly dissimilar (Bray-Curtis dissimilarities *Adonis* test p-value = 0.0005), with tissue and stool communities from the same infant sharing little overlap **(Fig 4)**.

**Fig 5:**
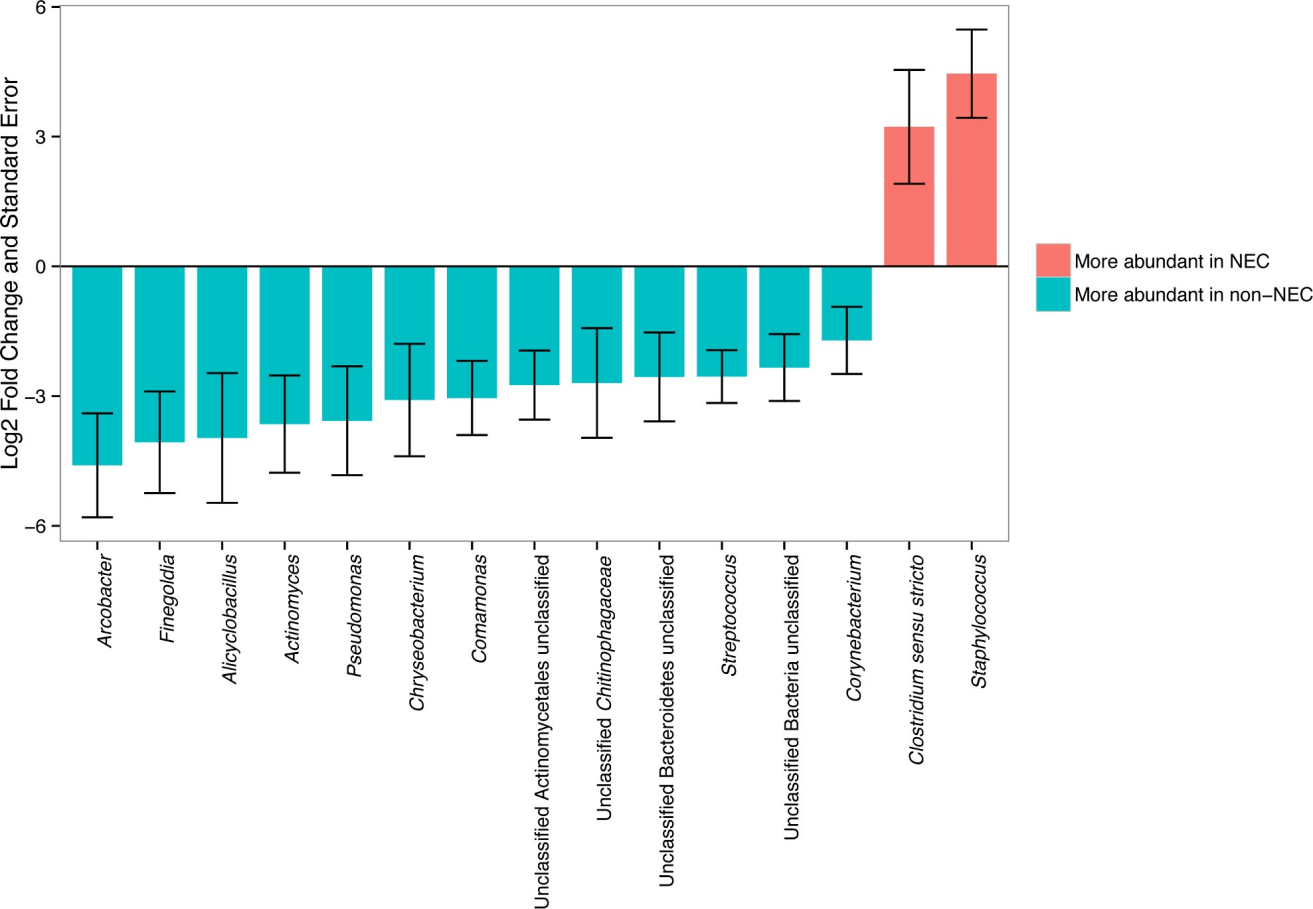
Comparison of the abundance of tissue bacterial genera between infants with and without necrotizing enterocolitis (NEC). Only bacterial genera that were significantly different between groups after adjusting for multiple comparisons using the DESeq2 package (see text for details) are indicated by an asterisk. Log_2_ fold change and log_2_ fold change standard error of tissue bacterial genera according to NEC status as calculated with the DESeq2 analysis. A log_2_ fold change of >0 (pink bars) indicates that abundance was detected to be higher during NEC, while a log_2_ fold change <0 (blue bars) indicates that abundance was detected to be higher in infants without NEC.

When tissue samples were analyzed alone, 15 taxa at the genus level had differential abundances in NEC compared to non-NEC samples with DESeq2 test q-values < 0.1 (**Fig 5**). *Staphylococcus* was ranked first in the DESeq2 model as most significantly different between NEC and non-NEC samples. *Clostridium_sensu_stricto* was near significantly more abundant in NEC tissue compared to non-NEC tissue (**Table 2**). Both groups were the only two genera identified as being significantly or near significantly more abundant in NEC compared to non-NEC samples. *Clostridium_sensu_stricto* was significantly more abundant in NEC than non-NEC tissues when the GeneSelector test using Wilcoxon test rankings was applied (q-value = 0.021). *Clostridium_sensu_stricto* abundance being higher in NEC infants appears to be due mostly to a single infant, N27, who had nearly 100% *Clostridium_sensu_stricto* abundance; the relative abundance of this genus was low for the remainder of samples (**Fig 4**).

**Table 2:** Difference in the abundance of selected tissue and fecal genera or OTUs in infants for a number of tested comparisons for taxa that were significant with the DESeq2 test.

Taxon	Sample type	Groups compared	Base mean*	Log_2_ fold change^†^	q-value^‡^
*Staphylococcus*	tissue	NEC/non-NEC	177.975	4.455	0.0006
*Clostridium sensu stricto*	tissue	NEC/non-NEC	1.552	3.226	0.0519
*Staphylococcus*	fecal	NEC/non-NEC	317.6	4.696	0.0002
*Staphyloocccus*.OTU0004	fecal	NEC/non-NEC	230.90	5.32	6.26e-04
*Staphyloocccus*.OTU0004	tissue	NEC/non-NEC	148.7	4.703	0.001
*Actinomyces*	tissue	NEC/non-NEC	1.550	-3.647	0.008
*Corynebacterium*	tissue	NEC/non-NEC	1.960	-1.711	0.087
*Staphylococcus*	tissue	Male/female	78.701	-3.699	0.012
*Actinomyces*	fecal	Age (days)	7.725	0.0871	0.004
*Staphylococcus*	tissue	Vancomycin (yes/no)	78.701	3.758	0.015

* Base means are calculated in the DESeq2 package for each taxon after normalizing read counts for each sample to account for differences in sequencing depth.

^†^ Log_2_ fold changes are calculated by the DESeq2 package and indicate the magnitude of the difference in abundance for each comparison. For categorical tests, positive values indicate that DESeq2 estimated the taxon was more abundant in the first tested group while negative values indicate that DESeq2 estimated the taxon was more abundant in the second group. When age in days was used as the group to test, a positive value indicates that DESeq2 found that taxon increased in abundance with each age day.

^‡^ Reported q-values are the result of a Wald test with the Benjamini and Hochberg correction for multiple comparisons.

NEC, necrotizing enterocolit

When fecal samples were analyzed alone, while *Clostridium_sensu_stricto* did not differ in abundance between NEC and non-NEC samples, *Staphylococcus* as a genus was more abundant during NEC (**Table 2**), consistent with findings from a recent study describing fecal microbiome samples from NEC patients [42]. A single *Staphylococcus* OTU, identified as OTU0004, was dominated in NEC fecal samples (**Table 2**) compared to non-NEC samples. This same *Staphylococcus* OTU0004 was also found to be significantly more abundant in tissue samples in infants with NEC (**Table 2**) compared to those without NEC. Due to the limited read lengths obtained, this OTU could not confidently be classified below the genus level.

### NEC-associated changes in the microbiome were stronger than the influence of other measured potential confounders

Although infants in the NEC and non-NEC groups were similar demographically and had similar environmental exposures in aggregate (all p-values >0.05), we conducted additional analyses to assess the effect of potential gut microbiome confounders.

Mode of delivery did not have a significant correlation with infant microbiome richness, alpha or beta diversity, or abundance of specific taxa in either stool or tissue samples. Infant sex did not have a significant correlation with infant microbiome richness or alpha or beta diversity in either tissue or fecal samples; however, tissue from males had lower abundance of *Staphylococcus* (**Table 2**). Age of the infant at time of sampling was found to correlate with trends in the gut microbiome: overall, the microbial communities significantly differed between age groups (pairwise Bray-Curtis dissimilarities were calculated between each sample and infants were quartered into age groups of as even size as possible and the PermANOVA *Adonis* test was performed on these groupings; p-value = 0.011). We observed an association between microbial richness, diversity and infant age and this was strongly correlated with NEC status (**Fig 2**).

Prior to sampling, all but one infant had been exposed to antibiotics (**Table 1**). Of infants who had received antibiotics, all had received at least two different antibiotics and at least one broad-spectrum antibiotic. Half of all infants in this study (12/24) were treated with vancomycin. *Staphylococcus* abundance was higher in tissue taken from infants who had received vancomycin (**Table 2**). In contrast, *Staphylococcus* was not significantly more abundant in stool taken from infants with vancomycin exposure. Vancomycin exposure was not significantly associated with differential abundance of any other taxa or with alpha diversity or richness in either fecal or tissue samples.

**Fig 6:**
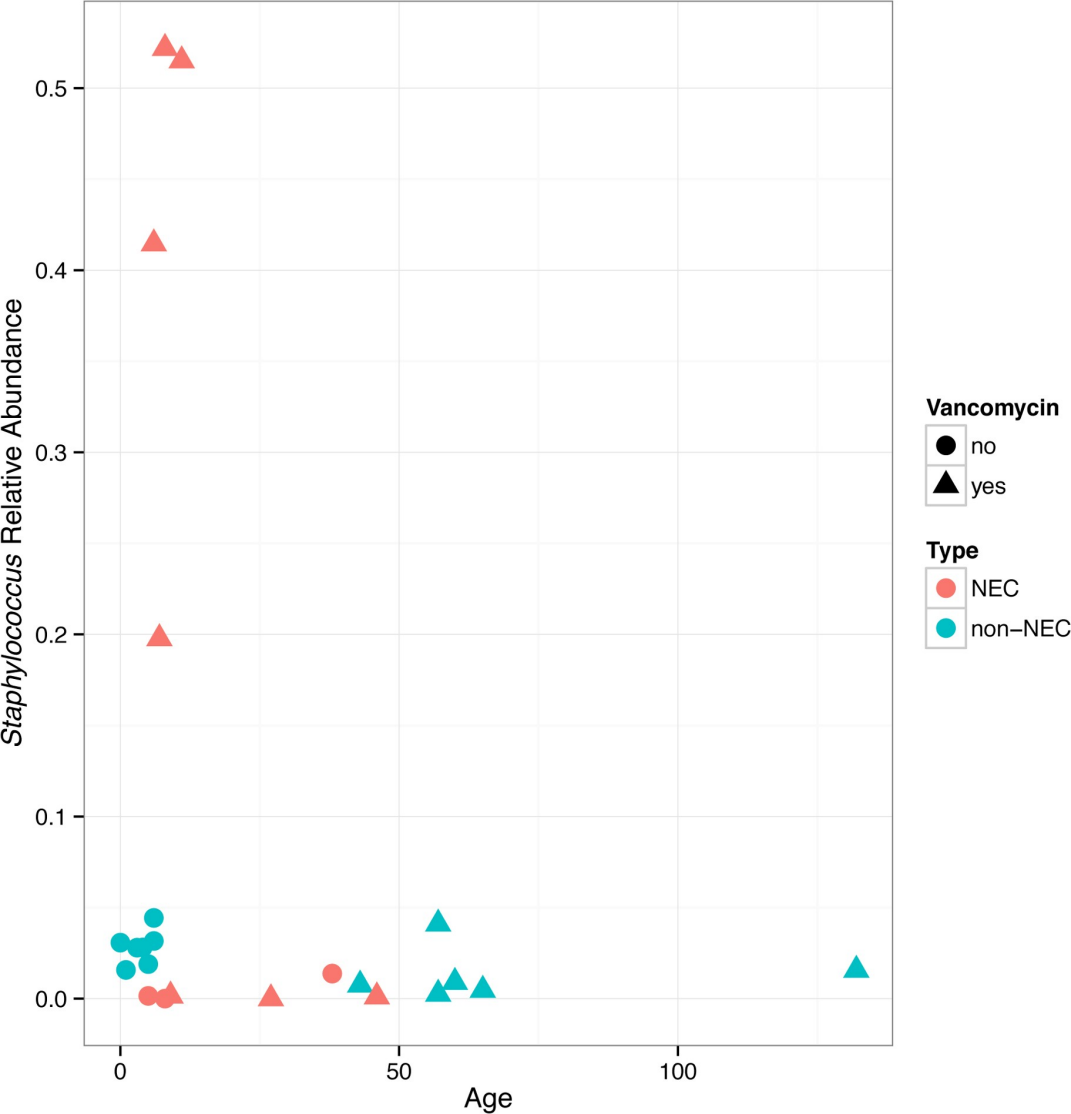
Age of the infant in days at time of sample collection is plotted on the x-axis and *Staphylococcus* relative abundance is plotted on the y-axis. Tissue *Staphylococcus* abundance is highest in infants with early necrotizing enterocolitis (NEC) resection, who received vancomycin.

To further assess the influence of confounding variables on the effect of NEC on the microbiome, we built models in DESeq2 to explicitly account for *a priori* selected covariates that may affect the gut microbiome (delivery mode, infant sex, infant age, diet, tissue type, exposure to vancomycin). Regardless of covariates added, the DESeq2 calculated log_2_ fold change in *Staphylococcus* abundance between infants with and without NEC directionality did not change (i.e., *Staphylococcus* abundance was always higher in NEC infants). Infant age and exposure to vancomycin had the strongest effect on the association between NEC on *Staphylococcus* abundance: young infants with NEC who had been exposed to vancomycin generally had high *Staphylococcus* tissue abundance (**Fig 6A**).

For ethical reasons, intestinal tissue cannot be collected from healthy infants; therefore, all the non-NEC infants in this study were in the hospital for ailments unrelated to NEC. There were two disparate groups of non-NEC infants recruited for this study: those who were very young (<1 week old) and those who were older (40+ days). To assess the impact of these two disparate non-NEC groups, we repeated our main analyses, after separating out the samples into three groups: 1) NEC 2) “young” non-NEC and 3) “old” non-NEC. A PCoA plot constructed using pairwise Bray-Curtis dissimilarities at the genus level revealed that the two non-NEC groups clustered together, separately from samples from infants with NEC (**S1 Fig**). Additionally, microbial richness was lowest in infants who had NEC and was similar in the two non-NEC groups, regardless of infant age (**S2 Fig**). *Staphylococcus* abundance was highest in the NEC group, followed by the youngest non-NEC group, and lowest in the older non-NEC group suggesting intestinal *Staphylococcus* colonization at early age.

### PICRUSt analyses reveals different functional profiles in NEC compared to non-NEC samples

**Fig 7:**
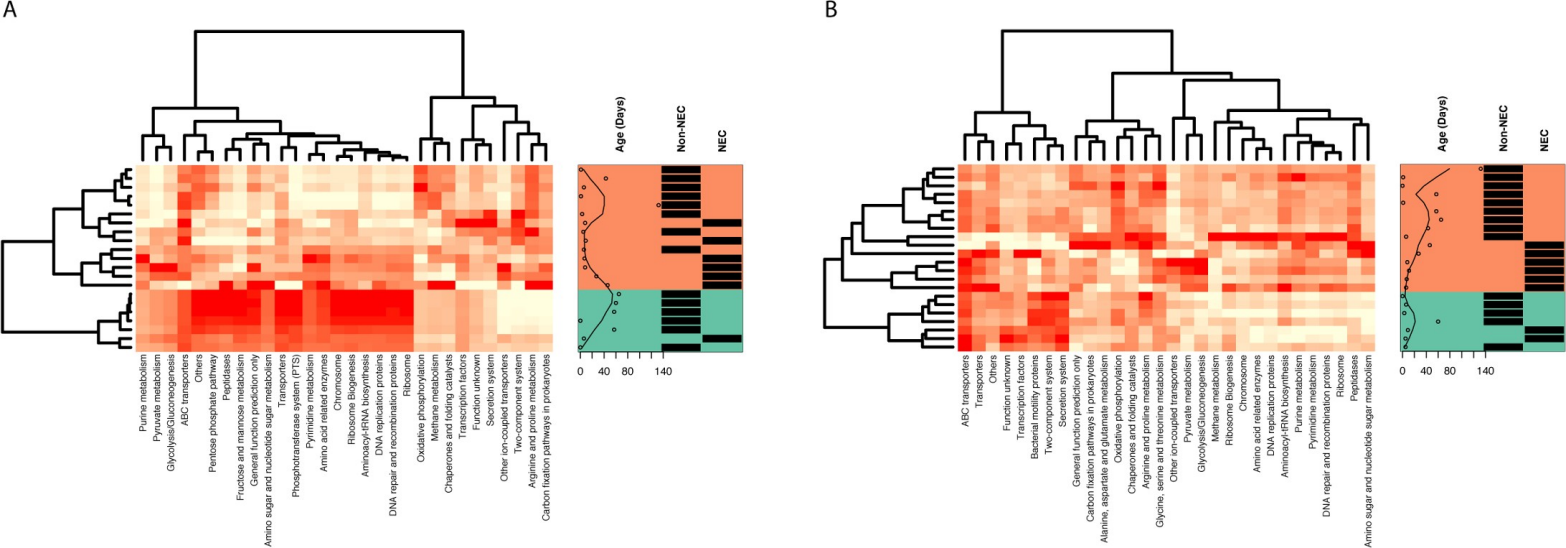
Phylogenetic Investigation of Communities by Reconstruction of Unobserved States (PICRUSt) was used to predict metagenomic and functional composition of the samples from 716S rRNA sequences. Heatmaps of normalized counts of microbial function pathways detected with the PICRUSt pipeline for infant A) stool and B) tissue are shown. Infant age, in days, and necrotizing enterocolitis (NEC) status are labelled for each sample. Both sample types display clustering associated with NEC status.

Overall, the predicted functional profile in NEC and non-NEC samples were generally distinct in stool (**Fig 7A**) and tissue samples (**Fig 7B**). Processes and pathways related to signatures of infectious diseases, i.e., bacterial toxins (base mean = 661.6, log_2_ fold change = 1.049, q-value = 2.045e-05) and *Staphylococcus aureus* infection (base mean = 831.7, log_2_ fold change = 0.724, q-value = 1.209e-02) were enriched in NEC tissue samples compared to non-NEC tissues.

## Discussion

Only a few studies have interrogated the tissue-level intestinal microbiome in NEC, despite the relative proximal location of intestinal injury and previous reports on the existence of a site-specific intestinal microbiome [13,43,44]. Here, we report a tissue-specific overrepresentation of Firmicutes, specifically *Staphylococcus sp*. and *Clostridium sp*. in NEC. We are aware of only two other reports on the NEC tissue-level microbiome in humans: a study from Denmark performed a retrospective analysis of formalin-fixed and paraffin-embedded tissue specimens using fluorescent in situ hybridization with bacterial rRNA-targeting oligonucleotide probes [45]. They detected Proteobacteria (49.0%), Firmicutes (30.4%), Actinobacteria (17.1%) and Bacteroidetes (3.6%) in tissue samples. More recently Brower-Sinning et al. applied 16S rRNA technology to compare the microbiome of 16 cryopreserved NEC samples and 10 controls [46]. Except for a higher bacterial load in NEC tissues, no statistically significant distinction was found between the composition of NEC and non-NEC microbial communities. The different results in our study may be explained by the fact that in the work by Brower-Sinning et al. all but one control patient were former NEC patients. In contrast, we included samples from infants with no history of NEC.

While we observed that the infant gut microbiome was significantly dissimilar in infants with NEC compared to those without NEC, we conducted a number of analyses to test the influence of potential confounders. After adding multiple covariates to the model in DESeq2 suggests that a combination of variables is likely to influence the infant tissue microbiome, for example age, vancomycin exposure, and NEC were found to correlate with *Staphylococcus* abundance. We observed that very young infants with NEC who had been exposed to vancomycin were most likely to have high *Staphylococcus* abundance in their gut tissue. We do not know how to explain this unexpected finding except by the fact that vancomycin does not penetrate tissue very well. Delivery by C-section has been associated with colonization of the neonate with *Staphylococcus* [47]. Therefore, we were surprised by our finding that mode of delivery did not correlate with specific taxa in our dataset. However, three out of four samples with high abundance of *Staphylococcus* were from C-section-delivered infants indicating that our sample size may have been insufficient to detect a statistical significance.

One unique aspect of our study is the direct comparison between tissue and fecal samples. This allows for an additional level of quality control as each patient is his/her own control and results between fecal and tissue samples were distinct in both non-NEC and NEC patients. Consistent with previous studies in preterm infants [9,48,49], we confirmed the dominant phyla as Proteobacteria and Firmicutes, with a smaller contribution (<20%) from Bacteroidetes and Actinobacteria. Several fecal microbiome studies reported a bloom of γ-Proteobacteria with a concomitant decrease in Firmicutes in NEC patients [10,12]. This shift in microbial communities in NEC patients appears to start 1–2 weeks prior to diagnosis and has been associated with metabolic changes [9]. While our data do not replicate this shift in Proteobacteria in fecal samples, possibly as we measured the gut microbiome during rather than prior to NEC diagnosis, we confirmed the previously reported reduced microbial diversity and loss of Actinobacteria in NEC patients, especially patients with severe (surgical) disease [10,50].

Given numerous previous reports on the dominance of Proteobacteria in NEC [9,10], we were surprised to find the high prevalence of Firmicutes and specifically *Staphylococcus sp*. in NEC tissue. However, different forms of dysbiosis have been reported in NEC [11,48] including recently an association between *Clostridium* and *Staphylococcus* with NEC in European preterm infants [42]. Importantly, NEC dysbiosis with Firmicutes including *Staphylococcus* has been associated with earlier disease and higher mortality [48]. Our study included only infants with surgical NEC, the group of patients with highest mortality [51]. When comparing NEC patients with heavy versus light *Staphylococcus* abundance, NEC patients with high abundance required surgical resection significantly earlier. *Staphylococcus* is the major colonizing organism of the infant gut shortly after birth [49,52,53]. In preterm neonates, culture-based studies detected *Staphylococcus* in 50% of meconium and 100% of fecal samples from the first week post-partum [54]. *Staphylococcus sp*. are frequently cultured from meconium and have been associated with increased risk for NEC [8,55].

Our study has limitations. While we collected tissue and fecal samples prospectively, technical and ethical limitations do not allow for tissue sampling prior to surgical resection. Therefore, we cannot perform time series experiments to evaluate the dynamic microbiome changes in NEC tissue. Similarly, since it is currently not possible to sample intestinal tissue from normal infants, we lack a healthy control cohort in which to characterize the standard infant tissue microbiome. While we attempted to match for important variables such as mode of delivery, antibiotic exposure and type of feeding, given the nature of this human study that explores both tissue and stool of a surgical emergency in a very vulnerable population, we were not able to control for all possible microbiome confounders. In addition, the lack of shotgun metagenomic sequencing prohibits further classification of the bacteria, especially those of important genera e.g., *Staphylococcus* and *Clostridium* identified in this study. However, based on the recent findings by Rozé et al, we speculate that the majority of *Staphylococcus* and *Clostridium* species would be *S*. *aureus and C*. *neonatale* [42]. Future studies implementing whole genome sequencing will be necessary to address strain identification and implications for derangements in metabolic function associated with the distinct microbial community structure we detected. An additional future aim could be to measure *Staphylococcus*-specific endotoxin production in stool samples, especially as our PICRUSt data suggests there was an increase in bacterial toxin pathways in NEC compared to non-NEC tissue samples.

## Conclusion

To the best of our knowledge, we define here for the first time corresponding fecal and tissue-level microbial communities comparing NEC patients with patients without a history of NEC and confirm age and antimicrobial exposure as defining factors.

## Supporting information

S1 Fig Principal coordinates analysis (PCoA) plots of tissue samples, labelled by necrotizing enterocolitis (NEC) status and whether the infant was from the young or old non-NEC group. Bray-Curtis dissimilarities between samples were calculated at the OTU level after normalizing read counts to simple proportions. NEC and non-NEC samples are observed to cluster separately, while both the young and old non-NEC samples clustered together.
(TIF)

S2 Fig Microbial richness was estimated using two indices, the Chao estimator (S.chao1) and estimated number of operational taxonomic units (OTUs) (S. obs) and each of these indices was plotted as a function of infant age in days. Two age disparate groups of non-necrotizing enterocolitis (NEC) infants were included in the analysis; however, from this figure it can be observed that NEC/non-NEC status had a much stronger effect on microbial richness than infant age.
(TIF)
