# Supplementary figures and images for: Distinct mucosal microbial communities in infants with surgical necrotizing enterocolitis correlate with age and antibiotic exposure
Source: PLoS One. 2018 Oct 26;13(10):e0206366. doi: 10.1371/journal.pone.0206366 (PMC6203398; doi:10.1371/journal.pone.0206366)

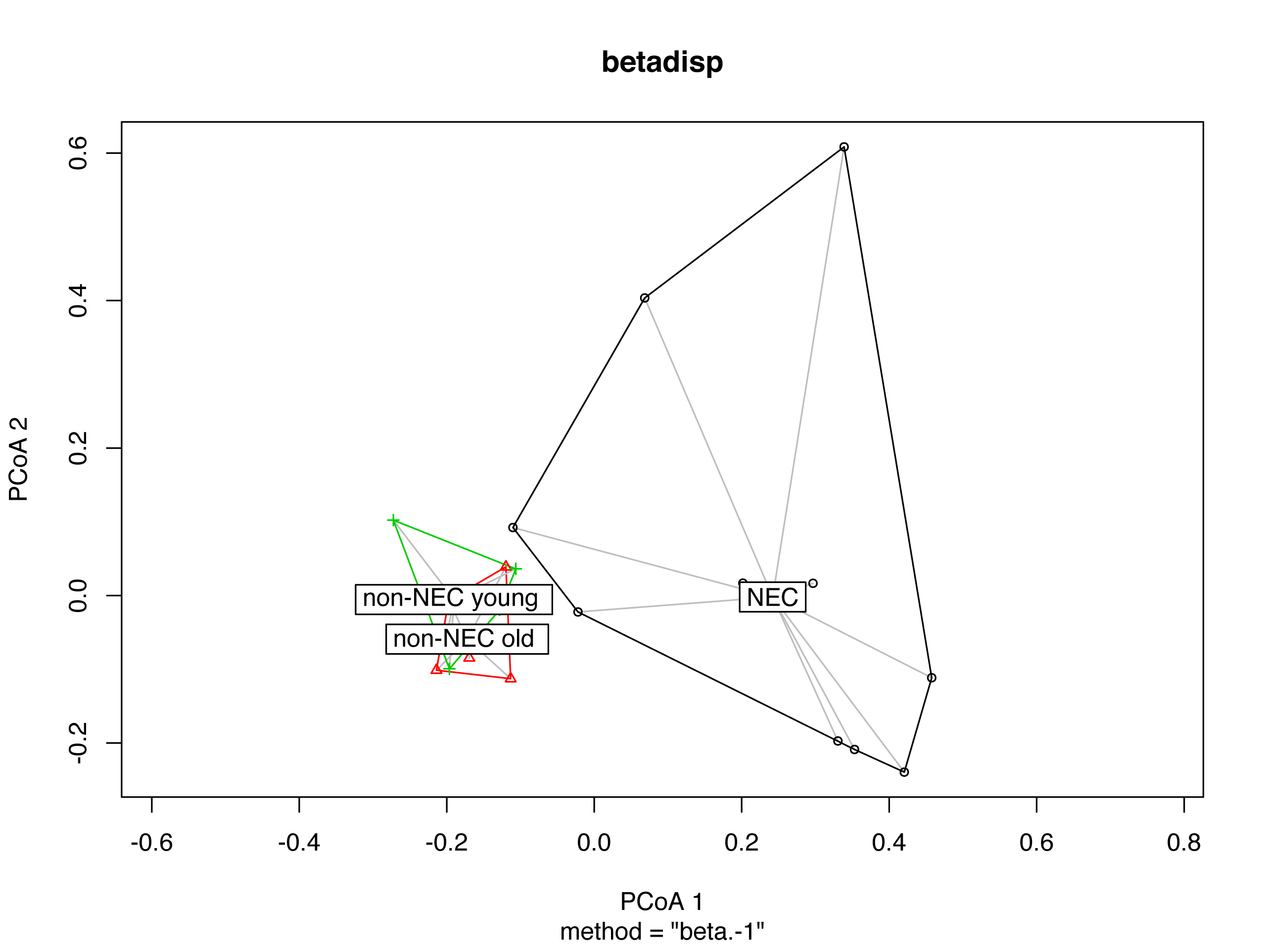

Supplement: S1 Fig — Bray-Curtis dissimilarities between samples were calculated at the OTU level after normalizing read counts to simple proportions. NEC and non-NEC samples are observed to cluster separately, while both the young and old non-NEC samples clustered together. (TIF) [file pone.0206366.s001.tif]

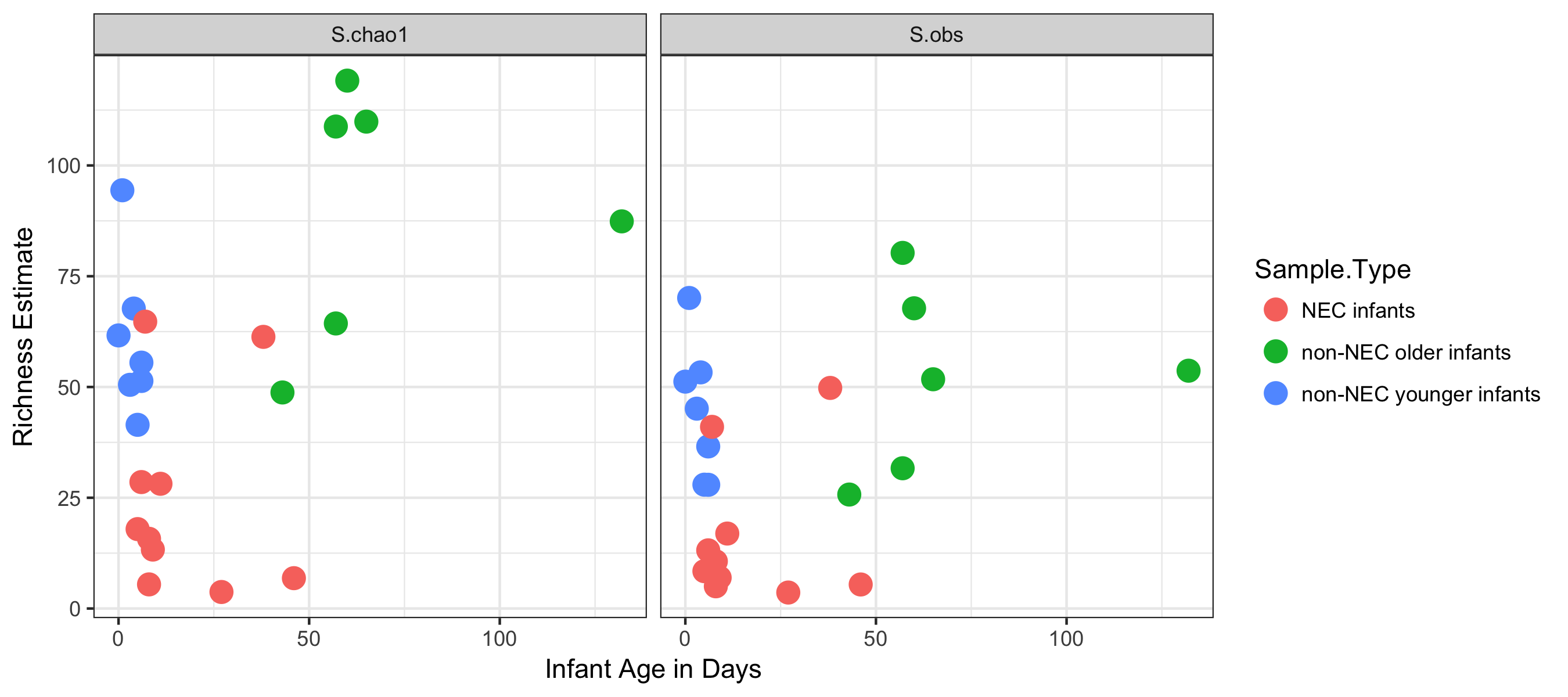

Supplement: S2 Fig — Two age disparate groups of non-necrotizing enterocolitis (NEC) infants were included in the analysis; however, from this figure it can be observed that NEC/non-NEC status had a much stronger effect on microbial richness than infant age. (TIF) [file pone.0206366.s002.tif]
